# Modelling Between-Cell-Type Heterogeneity in Within-Host Influenza Virus Infection

**DOI:** 10.1007/s11538-026-01759-4

**Published:** 2026-09-28

**Authors:** Ada W. C. Yan, Steven Riley, James M. McCaw

**Affiliations:** 1 Department of Mathematical and Geospatial Sciences, RMIT University, Melbourne, Victoria Australia; 2 Melbourne School of Population and Global Health, The University of Melbourne, Parkville, Victoria Australia; 3 Department of Infectious Disease, Imperial College London, London, United Kingdom; 4 School of Public Health, Imperial College London, London, United Kingdom; 5 School of Mathematics and Statistics, The University of Melbourne, Parkville, Victoria Australia

**Keywords:** Viral dynamics, Reproduction number, Transmissibility, Identifiability

## Abstract

Cell tropism, or the preference of a virus for particular cell types, has major implications for viral transmission, pathogenesis, and evolution. Historically, changes in cell tropism for particular viruses have been observed to correlate to changes in viral fitness, in the form of increased within-host replication and increased transmission between hosts. This is illustrated in the context of influenza, where adaptation to infect cells expressing α2-6 linked sialic acid receptors enhances human-to-human transmissibility. Target cell populations differ not only in abundance but also in intrinsic properties such as susceptibility, viral production, and interferon responses, rendering the relationship between tropism and viral fitness multi-faceted and complex. Understanding how different cell tropisms quantitatively change fitness remains an important open question in virology and quantitative biology. Here, we present a within-host mathematical model that incorporates distinct target cell types differing in key properties, and examine how cell tropism affects fitness-related summary statistics such as peak viral load, infection duration, or total virus produced. Our analysis reveals that tradeoffs may arise when cell types differ by multiple characteristics. We further demonstrate that model parameters describing heterogeneity between cell types can be more accurately inferred when cell type proportions are measured alongside viral load. Our findings provide a framework for assessing the links between viral evolution, cell tropism, and within-host fitness, and motivate the design of experiments to collect quantitative data on between-cell-type heterogeneity.

## Introduction

When a virus infects a host, different cell types are available to be infected. These cell types can differ in their susceptibility to infection, the amounts of (viable and non-viable) virus they produce, production of interferon, and ability to enter an antiviral state. Cell tropism refers to the preference of a virus to infect one cell type over another. In some important cases, cell tropism is key to the fitness of a virus, defined as the ability of a virus to replicate within a host and cause onward transmission. For example, influenza viruses that cause pandemics have historically been of avian origin (with swine as an intermediate host in the case of the 2009 pandemic virus). In order to efficiently transmit between humans, influenza viruses need to evolve to preferentially infect cells which express α2-6 linked sialic acid receptors, rather than cells which express α2-3 linked sialic acid receptors that predominate in the reservoir species (Lipsitch et al. 2016). Non-ciliated cells, which express α2-6 linked sialic acid receptors, are more abundant in the human upper respiratory tract compared to ciliated cells which express α2-3 linked sialic acid receptors. For example, in the nasal cavity, only 20% of cells are ciliated, whereas in the bronchi branching into the lower respiratory tract, there are up to 90% ciliated cells (Yoshida et al. 2022; Sikkema et al. 2023). Therefore, it is plausible that a fitness advantage is conferred simply by adapting to infect more abundant cells at the initial site of infection. However, these cell types may also behave differently upon infection, such that the relationship between cell tropism and fitness becomes more complicated. For example, Roach et al. (2024) showed that when cell cultures with a mixture of cell types were infected with the same human influenza virus, a higher proportion of ciliated cells was associated with increased virus production. This result appears to contradict the observation that human influenza viruses must be adapted to infect non-ciliated cells, and suggests that the fitness advantage conferred by cell tropism could be more complicated, possibly due to cell-type-specific pro- or antiviral factors such as the innate immune response.

We wish to understand from a quantitative perspective: how do changes in viral fitness result from the evolution of viruses to infect different cell types? Given the characteristics of different cell types, is there always an optimal cell type to infect, or can there be tradeoffs such that infecting a similar proportion of each cell type is the optimal strategy? Many experiments in the scientific literature have been conducted where cells have been harvested from *in vitro* or *in vivo* infections, and analysed using techniques such as single-cell RNAseq (Fiege et al. 2021; Kelly et al. 2022; Lindeboom et al. 2024; Yoshida et al. 2022; Sikkema et al. 2023; Woodall et al. 2024) or flow cytometry (Roach et al. 2024). We would like to use these data to answer the proposed questions.

Within-host mathematical models have been used to quantify mechanisms of infection and immunity, and understand how these mechanisms contribute to the time course of infection. A number of mathematical models incorporating different types of target cells (cells available for infection) have been constructed and analysed in the literature. Many of these models are for chronic infection with pathogens such as HIV, so the focus of these analyses have been on the stability of equilibria (Elaiw 2010). Stability analysis for models of acute infection with multiple equilibria have also been studied (Payne et al. 1992; Wang et al. 2016). However, there are a small number of studies of the transient dynamics of infections (Perelson et al. 1997; Dahari et al. 2005; Bajaria et al. 2002). Some models include different spatial locations for infection (Higgins et al. 2025) and model the spread of virus between locations.

An important problem spanning many such models, and a ubiquitous challenge for within-host mathematical modelling, is that these models have many parameters, and it is unclear that their values can be estimated accurately from data.

In this study, we formulate a within-host viral dynamics model where cell types differ in their susceptibility to infection, amount of virus produced, and/or production of interferon. We show that changing the relative susceptibility to infection of the different cell types can alter summary statistics for various parts of the virus replication cycle, which contribute to viral fitness in different ways. We show that there can be tradeoffs in the optimal cell type to infect, for example if the cell type that produces the most virus also produces the most interferon. We then show that model parameters related to the degree of heterogeneity can be inferred if data on the proportion of cells of each type are available in addition to viral load data.

## Methods

### Model

We consider a simple model for acute viral infections, incorporating innate and adaptive immunity:

1 $$\begin{aligned} \begin{aligned} \frac{dT}{dt}&= -\beta TV - \phi FT + \rho R,\\ \frac{dR}{dt}&= \phi FT - \rho R,\\ \frac{dI}{dt}&= \beta TV - \delta I,\\ \frac{dV}{dt}&= p I - (c + k A) V,\\ \frac{dF}{dt}&= qI - dF,\\ \frac{dA}{dt}&= mA \left( 1 - \frac{A}{A_{max}}\right) . \end{aligned} \end{aligned}$$

with initial conditions $T(0) = T_0$, R(0)=0, I(0)=0, $V(0) = V_0$, F(0)=0, $A(0) = A_0$.

This model draws upon models by Cao et al. (2015) and Handel et al. (2007). It includes the following processes:

- Target cells (*T*) become infected at rate βV;
- infected cells (*I*) produce free virus (*V*) at rate *p*;
- infected cells produce interferon (*F*) at rate *q*;
- free virus is neutralised by antibodies (*A*) at rate *k*;
- interferon renders target cells resistant to infection (*R*) at rate ϕ;
- resistant cells revert to being susceptible at rate ρ;
- virus, infected cells, and interferon decay at rates *c*, δ and *d* respectively;
- antibodies are not modelled mechanistically, but they are assumed to grow exponentially at rate *m* from the start of infection onwards, with a carrying capacity $A_{max}$.

Note that *V* refers to free infectious virus (e.g. measured by TCID_50_ or plaque assay) rather than genome copy number (e.g. measured by RT-qPCR). While many different mathematical forms for the innate and adaptive immune responses in influenza virus infection have been proposed in the literature (e.g.Baccam et al. (2006); Smith et al. (2018); Pawelek et al. (2012); Cao et al. (2015); Miao et al. (2010)), we chose simple forms that are sufficient for the purposes of our study. Some immune mechanisms such as natural killer cells and CD8+ T cells have not been included. The model structure and chosen parameter values are such that the innate immune response prevents target cell depletion and lowers the peak viral load, and that the adaptive immune response, rather than target cell depletion, is responsible for resolution of the infection.

**Fig. 1:**
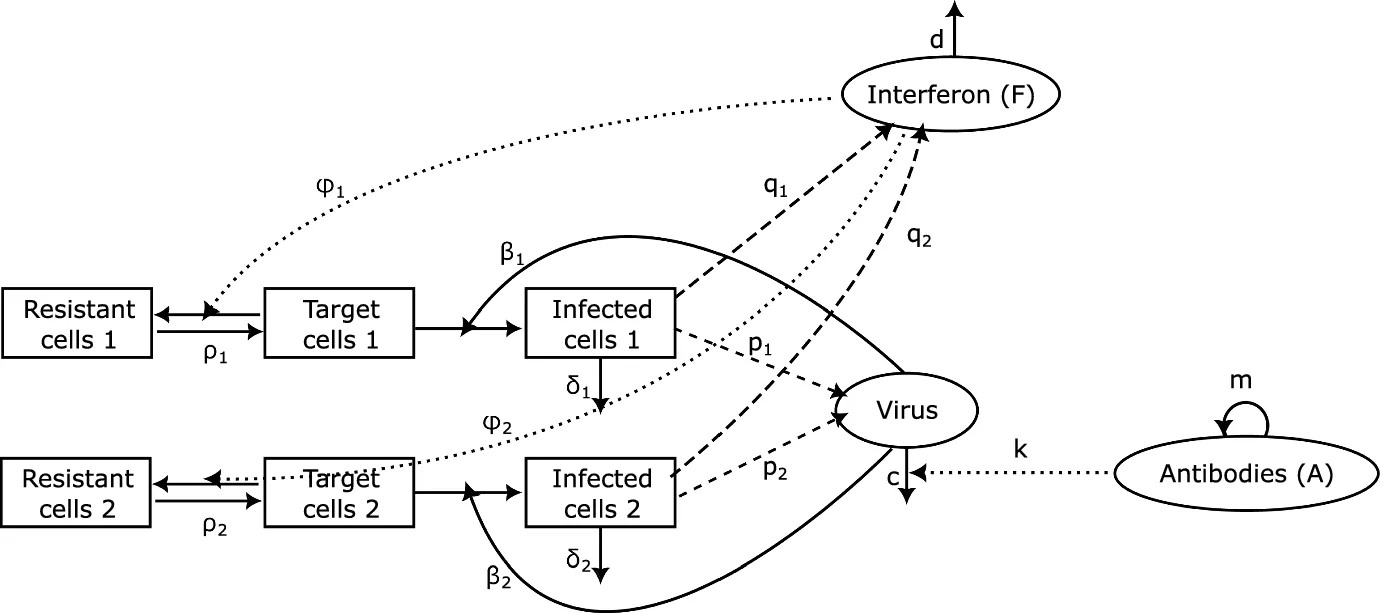
Compartmental model in Eq. 2 for N=2 cell types

With *N* cell types, the model equations become

2 $$\begin{aligned} \begin{aligned} \frac{dT_i}{dt}&= -\beta _i T_i V - \phi _i FT_i + \rho _i R_i, \qquad i = 1, \dots , N\\ \frac{dR_i}{dt}&= \phi _i FT_i - \rho _i R_i,\\ \frac{dI_i}{dt}&= \beta _i T_i V - \delta _i I_i,\\ \frac{dV}{dt}&= \sum _{i=1}^N p_i I_i - (c + kA)V,\\ \frac{dF}{dt}&= \sum _{i=1}^Nq_iI_i - dF,\\ \frac{dA}{dt}&= mA \left( 1 - \frac{A}{A_{max}}\right) . \end{aligned} \end{aligned}$$

with initial conditions $T_i(0) = T_{0i}$, $R_i(0) = 0$, $I_i(0) = 0$, $V(0) = V_0$, F(0)=0, $A(0) = A_0$. Figure 1 shows the compartmental model in Eq. 2 for N=2 cell types.

Table 1 shows the baseline values for each parameter, based on those by Cao et al. (2015) and Handel et al. (2007). The parameter values were chosen such that the resulting viral load trajectories resembled those observed for *in vivo* influenza virus infections in ferrets. Specifically, the parameter values for β, δ, *p*, ϕ, ρ, *q*, $T_0$ and $V_0$ were taken directly from Cao et al. (2015). The antibody parameters *m*, $A_0$ and $A_{max}$ and viral decay parameter *c* were tuned such that the simpler mathematical form of the adaptive immune response produced similar quantitative results to Cao et al. (2015). Lack of parameter identifiability for viral dynamics models has previously been documented (Yan et al. 2019); there could potentially be other choices of parameter values producing realistic viral load curves, but we have chosen a single plausible parameter set for illustration of the model behaviour. Interferon and antibodies were quantified in relative units $u_F$ and $u_A$ as these compartments are often not directly measured over time.

**Table 1:** Baseline parameter values

Parameter	Value	Unit
β	$5 \times 10^{-5}$	$\textrm{TCID}_{50}^{-1}\textrm{d}^{-1}$
δ	3	$\textrm{d}^{-1}$
*p*	0.35	$\textrm{TCID}_{50}\textrm{cell}^{-1}\textrm{d}^{-1}$
*c*	4	$\textrm{d}^{-1}$
ϕ	0.14	$u_F^{-1}\textrm{d}^{-1}$
ρ	0.05	$\textrm{d}^{-1}$
*q*	$5 \times 10^{-5}$	$u_F\textrm{cell}^{-1}\textrm{d}^{-1}$
*k*	1	$u_A^{-1}\textrm{d}^{-1}$
*m*	1	$\textrm{d}^{-1}$
$T_0$	$7 \times 10^7$	$\textrm{cell}$
$V_0$	1	$\textrm{TCID}_{50}$
$A_0$	0.34	$u_A$
$A_{max}$	$10^4$	$u_A$

### Viral Load Simulation and Fitness-Related Summary Statistics

We used odin 1.2.6 (Fitzjohn 2024) in R 4.4.0 (R Core Team 2024) to numerically solve Eq. 2 for N=2 cell types, changing the amount of heterogeneity in one or more parameters. The model captures cell tropism through having different susceptibilities to infection ($\beta _i$) for each cell type, so $\beta _i$ was varied for all simulations.

We also selected fitness-related summary statistics to conduct comparisons between viruses: the within-host basic reproduction number ($R_0$); the growth rate (*r*); and a third summary statistic designed to capture transmissibility. These fitness-related summary statistics relate to different parts of the virus replication cycle, thus contributing to the overall fitness. Both the within-host basic reproduction number ($R_0$) and growth rate (*r*) measure the ability of the virus to replicate within a single host. The basic reproduction number and growth rate can be calculated using the next generation matrix approach (Diekmann et al. 2010), linearising the ODEs around the disease-free equilibrium:

3 $$\begin{aligned} \begin{aligned} \frac{dI_i}{dt}&= \beta _i T_{0i} V - \delta _i I_i,\\ \frac{dV}{dt}&= \sum _{i=1}^N p_i I_i - (c + kA_0)V.\\ \end{aligned} \end{aligned}$$

The basic reproduction number for a within-host viral dynamics model is conventionally defined as the mean total number of secondary infected cells generated by a single initial infected cell in an pool of uninfected cells:

4 $$\begin{aligned} R_0 = \sum _{i=1}^N\frac{\beta _i T_{0i} p_i}{\delta _i (c + k A_0)} \end{aligned}$$

We note that the next generation matrix approach defines the time between infection of the first cell, through to producing a virion, and finally for a virion to infect a second cell, to be two generations. Thus, the $R_0$ calculated using the next generation matrix needs to be squared to yield the result in Eq. 4.

In the case that all $\delta _i = \delta$, i.e. the infected cell death rate is the same across all cell types, the growth rate is

5 $$\begin{aligned} r = \frac{-(c + kA_0+\delta ) + \sqrt{[\delta - (c + k A_0)]^2 + \sum _i\beta _iT_{0i}p_i}}{2} \end{aligned}$$

The third summary statistic, related to the amount of virus shed by a host, affects the ability of a virus to transmit to another host. A number of biological assumptions, each of which implies a different mathematical form for this summary statistic, have been adopted in the literature, as compared by Asher et al. (2023). For our primary analysis we consider the area under the viral curve (AUC), defined as

6 $$\begin{aligned} AUC = \int _0^\infty V(t) dt. \end{aligned}$$

We also consider a number of alternative relationships to map from viral kinetics to transmission (described and presented in the sensitivity analysis in Sec. 3.3). We note that a number of factors independent of viral load contribute to transmission. These include anatomical site and route of shedding, immune responses, symptom timing, and host behaviour, including contact patterns. A full multiscale treatment of transmission including these factors is beyond the scope of this study. Thus, when comparing transmissibility between different scenarios, we implicitly assume that the aforementioned factors remain the same between scenarios.

In this study, we have used viral titres in units of TCID_50_ as a scaled proxy for the number of infectious virions. The basic reproduction number is unitless while the growth rate has unit of 1/time; these two summary statistics are not affected by scaling of the viral load. The area under the curve (AUC) scales directly proportionally to any uniform scaling of the viral load, such that comparisons between AUCs are unaffected by the scaling.

### Simulation Estimation Study

We conducted a simulation-estimation study to investigate whether the degree of target cell heterogeneity can be inferred given different types of data, and how the identifiability of parameters with heterogeneity impacts the ability to make accurate model predictions. The number of combinations of parameters that could vary between cell types is large; we restricted the scope of this study to the case where the cell types differ in susceptibility ($\beta _i$) and abundance ($T_{0i}$).

#### Generating Parameter Sets for Simulation

For a model with two cell types (N=2) and heterogeneity in susceptibility to infection ($\beta _i$) only, to improve exploration of parameter space, we reparameterised $\beta _i$ in terms of the weighted mean of $\beta _i$, which we denote $\bar{\beta }$, and a parameter Δ which ranges between [-1,1] and controls the degree of heterogeneity between the two cell types. The equation for $\bar{\beta }$ is:

7 $$\begin{aligned} \bar{\beta } = \frac{\sum _i\beta _iT_{0i}}{\sum _iT_{0i}} \end{aligned}$$

For the homogeneous model with N=1, we have shown in previous work that the initial growth rate *r* is identifiable (Yan et al. 2019). Equation 5 shows that when there is only heterogeneity in $\beta _i$ and $T_{0i}$, *r* is a function of $\sum _i\beta _iT_{0i}$, rather than $\beta _i$ and $T_{0i}$ independently. Thus we expect $\bar{\beta }$ to be identifiable in a simulation-estimation study, justifying our choice of re-parameterisation.

We implicitly define Δ by the following functions to calculate $\beta _i$ given $\bar{\beta }$ and Δ:

8a $$\begin{aligned} \beta _1&={\left\{ \begin{array}{ll} \bar{\beta } (1 - \Delta T_{02}/T_{01}), & {\text {for}}\, \Delta < 0 \\ \bar{\beta } (1 - \Delta ), & {\text {otherwise}} \end{array}\right. }\end{aligned}$$

8b $$\begin{aligned} \beta _2&={\left\{ \begin{array}{ll} \bar{\beta } (1 + \Delta ), & {\text {for}}\, \Delta < 0 \\ \bar{\beta } (1 + \Delta T_{01}/T_{02}), & {\text {otherwise}} \end{array}\right. } \end{aligned}$$

This definition ensures that $\beta _1 = \beta _2$ for Δ=0; $\beta _2 = 0$, $\beta _1 = \bar{\beta } (1 + T_{02}/T_{01})$ for Δ=-1; and $\beta _1 = 0$, $\beta _2 = \bar{\beta } (1 + T_{01}/T_{02})$ for Δ=1. When Δ is thus defined, then when its value is 0, setting $\beta _1 = \beta _2$ and solving the ODEs in Eq. 2 leads to the same viral load as the homogeneous model, regardless of the values of $T_{0i}$ (assuming all other parameters are the same between compartments). The natural bounds for this definition of Δ (-1 and 1 when $\beta _1$ or $\beta _2$ are 0 respectively) are also the same for all values of $\beta _1$ and $\beta _2$. This definition thus enables us to compare the effects of heterogeneity across different parameter sets.

First, we used Latin hypercube sampling (implemented in *lhs* 1.2.0 Carnell 2024) to generate 5000 parameter sets, varying $\bar{\beta }$, δ, p/δ, *c* and $V_0$ in log space by one order of magnitude above and below the values given in Table 1, and $T_{01}/T_0$ in linear space between 0 and 1. The remaining parameters were held fixed. From the 5000 parameter sets, we selected those with realistic initial growth rates *r* (based on the homogeneous case, see Eq. 5) in the range $\ln (10^3) \le r \le \ln (10^5)$. As $V(t) \approx V(0) \exp (rt)$ in the initial phase of the infection, this restriction ensures that the viral load increases by 3–5 orders of magnitude during the first day of infection. 100 parameter sets (out of 558) fulfilling these criteria were randomly selected for subsequent analysis.

#### Simulating Data

For each of the 100 parameter sets, we solved the model system equations (Eq. 2) and generated simulated data for Δ=-0.8, Δ=0, and Δ=0.8. Simulated viral load data and cell proportion data were generated in triplicate for each parameter set.

We assume that cell proportion data are generated using common techniques such as flow cytometry or single-cell RNAseq. With these techniques, it is possible to distinguish infected from uninfected cells (using virus protein-specific antibodies for flow cytometry or proportion of viral RNA in a cell for single-cell RNAseq), and to distinguish different cell types (using antibodies for cell type markers for flow cytometry or cell type annotation for single-cell RNAseq). However, distinguishing between uninfected cells which are susceptible to infection (*T*) versus resistant to infection (*R*) is difficult. Hence, we define a variable for the number of uninfected cells, $U_i(t) = T_i(t) + R_i(t)$. Then, we generate cell proportion data by calculating the proportion of uninfected and infected cells of each type at a given time, out of total live cells, i.e., the data at time $t_i$ are

9 $$\begin{aligned} \begin{aligned} \textbf{D}(t) = [U_1(t_i)/M(t_i), U_2(t_i)/M(t_i), I_1(t_i)/M(t_i), I_2(t_i)/M(t_i)] \qquad {\text {where}} \\ M(t_i) = U_1(t_i) + U_2(t_i) + I_1(t_i) + I_2(t_i). \end{aligned} \end{aligned}$$

Cell proportion data were sampled on days 1 and 2 as the experimental methods to sample these data destroy the sample, limiting the number that can be taken. Each sample consists of a subset of the live cells at that time. We assume a sample size of 5000. The cell proportion data are assumed to follow a Dirichlet multinomial distribution with overdispersion parameter $\rho _0 = 0.01$, that is $[\hat{U_1}(t_i), \hat{U_2}(t_i), \hat{I_1}(t_i), \hat{I_2}(t_i)] \sim \textrm{DirMult}(C, \mathbf {\alpha })$ where $\mathbf {\alpha } = [U_1(t_i)/M, U_2(t_i)/M, I_1(t_i)/M, I_2(t_i)/M] \times (1 - \rho _0)/\rho _0$.

Viral load was sampled daily for seven days, with lognormal noise added to each replicate, such that $\log _{10}\hat{V} \sim \mathcal {N}(\log _{10}V, \sigma )$ where σ=0.5. An observation threshold was then imposed such that values under the lower limit of detection (10 TCID_50_) were censored.

#### Estimating Parameters

Once we sampled "true" parameter sets and simulated data using these parameter sets, we re-estimated the parameters from the simulated data to see if we could recover the true values. Inference was conducted using the No U-Turn sampler in Stan (Stan Development Team 2025) 2.37.0, with the interface cmdstanr (Gabry et al. 2025) 0.8.1. Using the default settings, eight parallel chains were sampled, with 1000 warm-up iterations and 1000 sampling iterations each. We expected some parameters to be unidentifiable, which is known to cause issues with convergence of chains, as well as divergent transitions in the No U-Turn sampler. We removed chains where divergent transitions were observed and chose a subset of chains (at least three), such that we retained as many chains as possible while keeping $\hat{R}$, a measure used to assess convergence, below 1.01. If this was not possible, we dropped the requirement for chains to have no divergent transitions, and chose the subset of chains (at least three) which minimised $\hat{R}$.

We estimated $\bar{\beta }$ and Δ either using viral load data only, or using both viral load and cell proportion data. All other parameters were assumed to be known, as we are focusing on the identifiability of parameters modelled to have heterogeneity. We set the prior distribution for $\bar{\beta }$ as uniform in log space ($\log _{10} \bar{\beta }\sim \mathcal {U}(-8, -4))$, and the prior distribution for Δ as uniform between -1 and 1.

We conducted a sensitivity analysis where *p* and δ were estimated in addition to $\bar{\beta }$ and Δ. Uniform priors were placed on $\log _{10}\left( p/\delta \right)$ and $\log _{10}(\delta )$. These priors were centred at the values in Table 1 and had a width of 2 (in log space).

Code to reproduce all results in the study, and all simulated data, can be found at https://github.com/ada-w-yan/fluheterogeneitypaper.

## Results

### The Model Captures Increases in Fitness-Related Summary Statistics When Preferentially Infecting Cells Which are More Abundant, Produce More Virus, or Produce Less Interferon

There are multiple reasons for why a change in cell tropism results in increased overall viral fitness. The increase could be due to the higher abundance of the newly preferred cell type (Lipsitch et al. 2016); an increased viral production rate (for example, through more efficient use of host proteins in replication and/or transcription Long et al. 2016); or a decreased rate of innate immune response induction.

In our modelling framework, we do not have a single measure for overall viral fitness; instead, we have defined three summary statistics relating to different parts of the viral replication cycle, which are positively correlated with overall fitness. We first confirm whether the model predicts increases in the summary statistics for these three scenarios:

(A) preferentially infecting cells which are more abundant;
(B) preferentially infecting cells which produce more virus;
(C) preferentially infecting cells which produce less interferon.

In all scenarios in this study, we simulate a changing cell tropism by varying $\beta _1$ and $\beta _2$ (susceptibility to infection of each cell type) while keeping their sum, $\beta _1 + \beta _2$, fixed. That is, we assume that when a virus evolves to change the cell type it prefers to infect, it increases the rate at which it infects cell type 1 by the same amount it decreases the rate at which it infects cell type 2 (or vice versa). In reality, virus evolution could result in arbitrary changes to $\beta _1$ and $\beta _2$, rather than necessarily evolving along a trajectory where $\beta _1 + \beta _2$ stays constant. However, if we consider the case where cell types are identical and equally abundant (same $p_i$, $\delta _i$, $q_i$, $\phi _i$, $\rho _i$ and $T_{0i}$), then changing $\beta _1$ and $\beta _2$ while keeping $\beta _1 + \beta _2$ constant conserves $R_0$ (by examination of Eq. 4). Thus, if $\beta _1$ and $\beta _2$ are changed while keeping their sum constant, any change in $R_0$ must be due to heterogeneities between cell types. Conversely, if $\beta _1$ and $\beta _2$ are allowed to change arbitrarily, changes in $R_0$ are partially attributable to a change in the mean susceptibility of cells had the cell types been otherwise identical and equally abundant. Thus, we focus on the scenario where $\beta _1$ and $\beta _2$ are varied while keeping their sum fixed.

In scenario A (unequal cell abundances), we set $T_{01} = 5.6 \times 10^7$, $T_{02} = 1.4 \times 10^7$, such that cell type 1 has an 80% abundance, similar to non-ciliated cells in the human upper respiratory tract (Yoshida et al. 2022; Sikkema et al. 2023). All other parameters are equal between cell types as per Table 1. For scenario B (unequal virus production rates), we set $p_1 = 0.56$, $p_2 = 0.14$, such that cell type 1 produces virus at a greater rate. Inferring production rates of virus from different cell types from existing data is less straightforward, so we have set that cell type 1 produces 80% of virus to keep the ratios the same as scenario A. All other parameters are equal between cell types. For scenario C (unequal interferon production rates), we set $q_1 = 2.5\times 10^{-5}$, $q_2 = 7.5 \times 10^{-5}$, such that cell type 1 produces interferon at a lower rate, and all other parameters are equal between cell types. While some choices in the degree of heterogeneity were arbitrary, the results are intended to show possible qualitative behaviours of the model and are not tied to particular choices of parameter values.

**Fig. 2:**
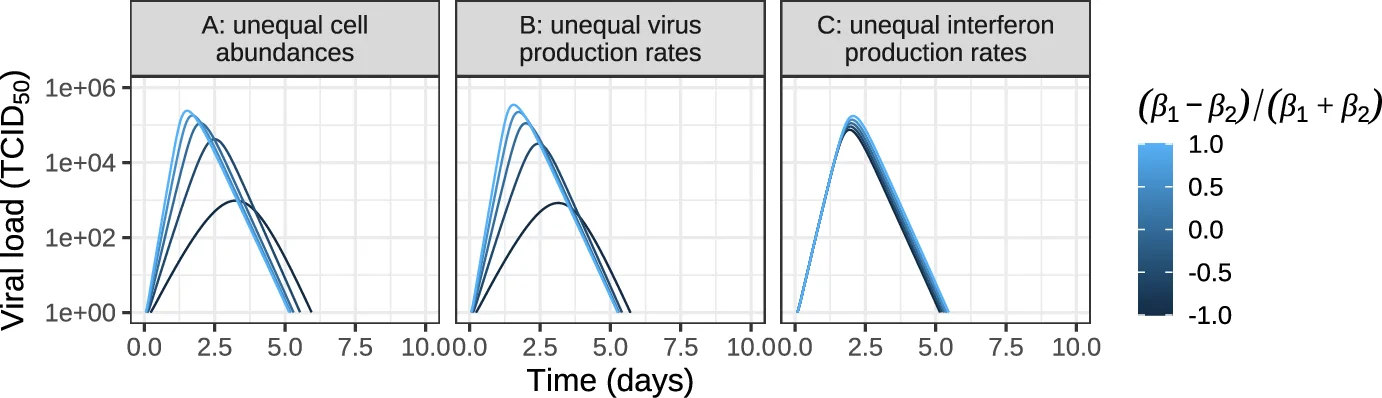
Simulated viral loads for scenarios **A** (unequal cell abundances), **B** (unequal virus production rates) and **C** (unequal interferon production rates) when $\beta _1 - \beta _2$ is changed (colours) while $\beta _1 + \beta _2$ is held constant

Figure 2 shows the simulated viral loads for Scenarios A, B and C as $\beta _1 - \beta _2$ is changed (colours) while $\beta _1 + \beta _2$ is held constant. That is, we see how the change in cell tropism leads to a change in viral load if cells have unequal abundance, virus production rates are unequal between cells, or interferon production rates are unequal between cells. In Scenarios A and B, the viral load increases more quickly initially for viruses preferentially infecting cell types that are more abundant (Scenario A) or that produce more virus (Scenario B). In Scenario C where the cell types differ by induction rate of interferon, the initial rate of increase of viral load is the same regardless of cell tropism. In all three scenarios, as expected, the peak viral load is higher for viruses preferentially infecting cell types that are more abundant (Scenario A), that produce more virus (Scenario B), or that produce less interferon (Scenario C). However, it is only in scenarios A and B that the initial growth rate is impacted by cell tropism. We now explore how this result relates to the change in within-host reproduction number.

**Fig. 3:**
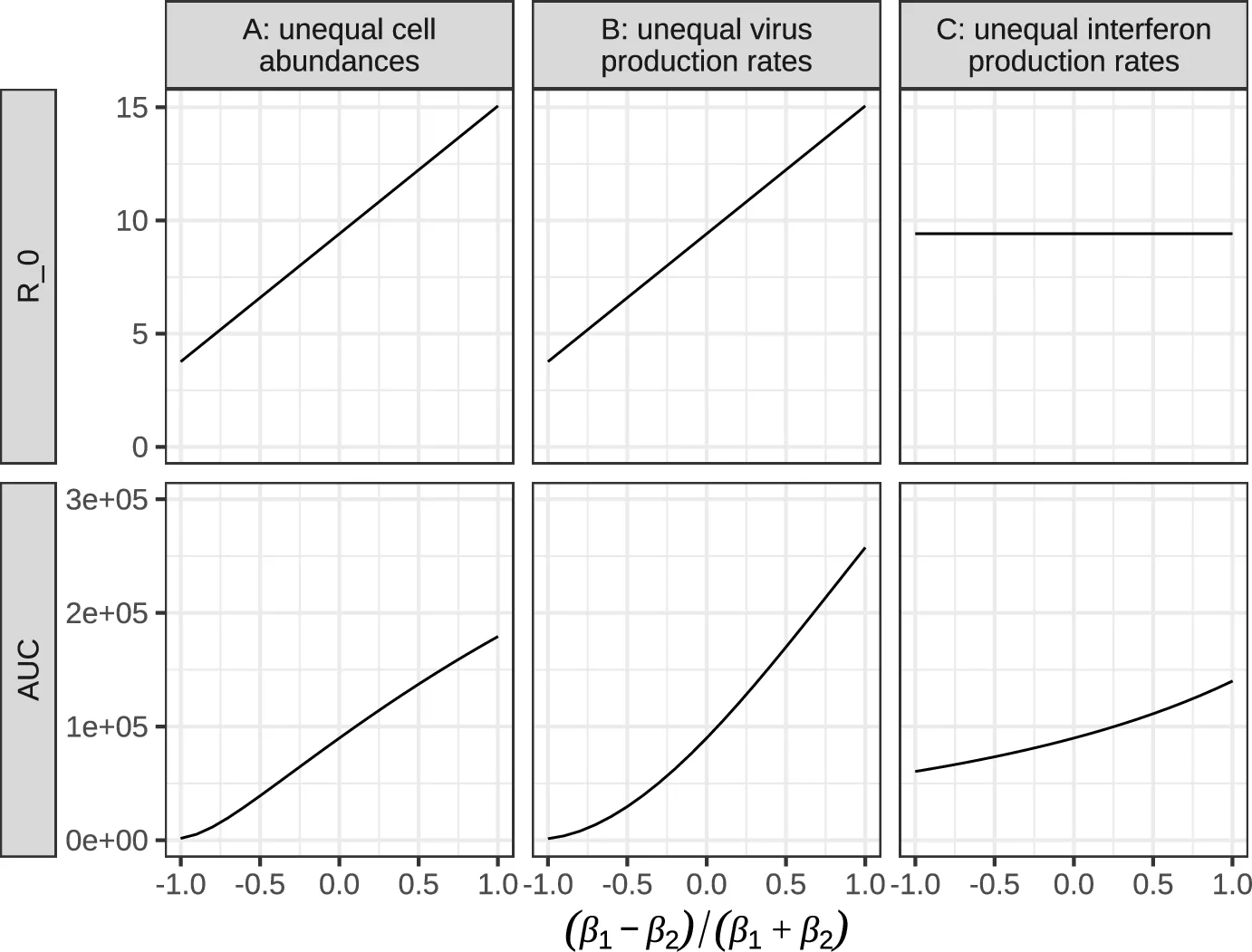
(Top) Basic reproduction number (bottom) AUC for scenarios **A**, **B** and **C** when $\beta _1 - \beta _2$ is changed (x-axis) while $\beta _1 + \beta _2$ is held constant

Figure 3 shows (top) the within-host basic reproduction number and (bottom) the AUC for scenarios A, B and C when $\beta _1 - \beta _2$ is changed (x-axis) while $\beta _1 + \beta _2$ is held constant. For Scenarios A (preferentially infecting cell types that are more abundant) and B (preferentially infecting cell types that produce more virus), increasing preference for cell type 1 increases the basic reproduction number, while for Scenario C (preferentially infecting cell types that produce less interferon), the basic reproduction number remains constant regardless of cell tropism. As the definition of the basic reproduction number pertains to secondary cells starting from a completely susceptible population of cells with no interferon, the changes in eventual interferon levels due to changed cell tropism do not change the basic reproduction number.

For a model with equal $\delta _i$, we can multiply both sides of Eq. 4 by $\delta (c + k A_0)$ and substitute into Eq.  5 to yield

10 $$\begin{aligned} r = \frac{-(c + kA_0+\delta ) + \sqrt{[\delta - (c + k A_0)]^2 + R_0 \delta (c + k A_0)}}{2} \end{aligned}$$

Thus, for fixed $c + k A_0$ and δ, *r* changes monotonically with $R_0$. In other words, a change in $\beta _i$, $p_i$ or $T_{0i}$ which increases $R_0$ also increases *r*, and vice versa. Therefore, the relationship between $\beta _1 - \beta _2$ and $R_0$ also holds for $\beta _1 - \beta _2$ and *r*.

For all scenarios, an increasing preference for cell type 1 increases the area under the viral load curve. Figure 1 in the Supplementary Material shows the viral load curves on the linear scale, to enable easier comparison with the AUC plots.

### Tradeoffs When Cells Differ in More than One Aspect

So far, we have considered cases where cells differ in abundance, viral production rate, or interferon production rate, in addition to susceptibility to infections. A natural question to consider, both from first principles and as a logical consequence of the above two results, is what would happen if the cells differ in more than one aspect other than tropism?

**Fig. 4:**
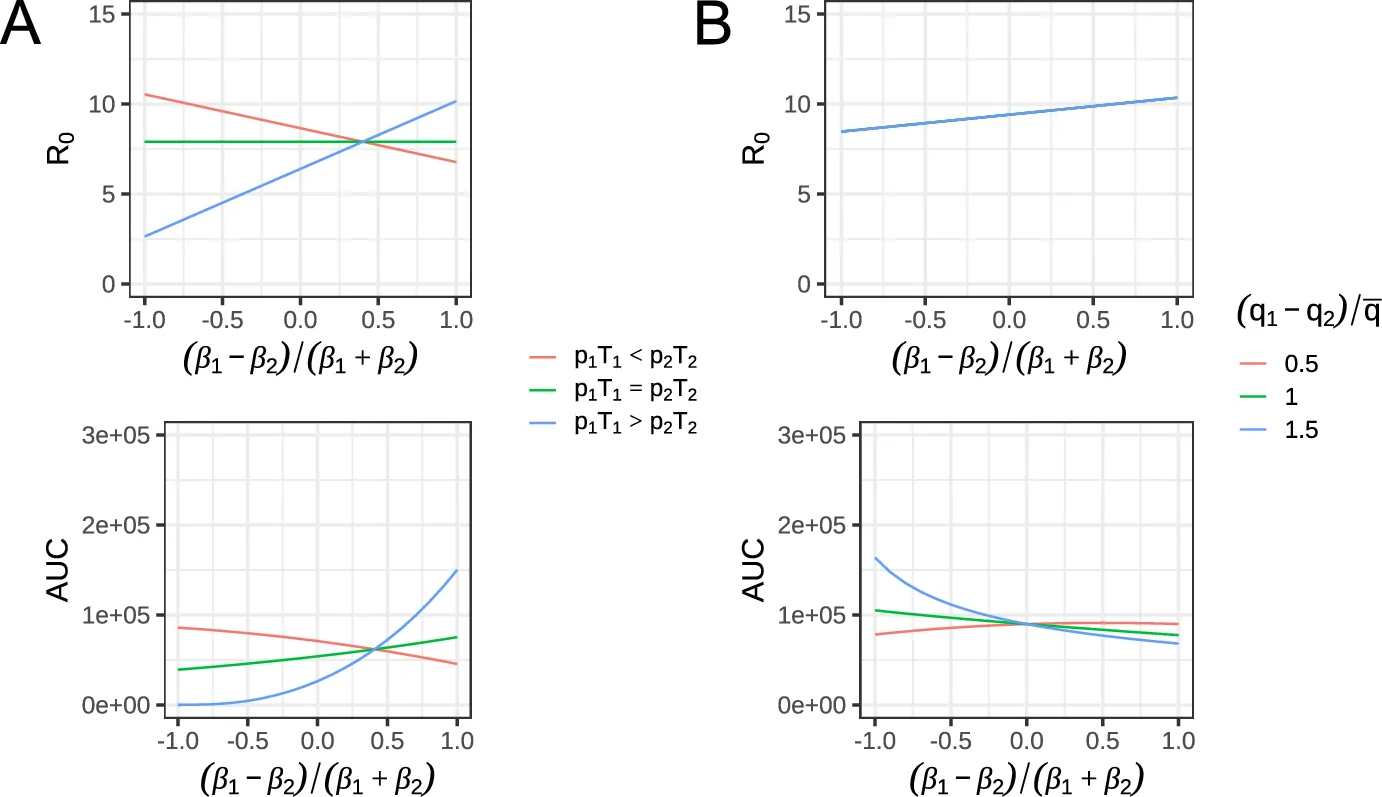
(Top) basic reproduction number and (bottom) area under the viral load curve (AUC) when **A** the cell type with a higher viral production rate is also less abundant than the other cell type; **B** the cell type with a higher viral production rate also has a higher interferon production rate

First, we use the model to predict how fitness-related summary statistics change with cell tropism when the cell type with a higher viral production rate is also less abundant than the other cell type (Fig. 4A). We fixed $T_{01} = 2.1 \times 10^7$, $T_{02} = 4.9 \times 10^7$ and simulate using three different sets of values for $p_i$: (i) $p_1 = 0.42, p_2 = 0.28$; (ii) $p_1 = 0.49, p_2 = 0.21$; (iii) $p_1 = 0.63, p_2 = 0.07$ TCID_50_cell^-1^d^-1^. For all three of these parameter sets, $p_1 > p_2$ and $T_{01} < T_{02}$. Inspection of Eq. 4 and numerical simulation (Fig. 4A) show that if $p_1 T_{01} = p_2 T_{02}$ (the case where $p_1 = 0.49, p_2 = 0.21$ TCID_50_cell^-1^d^-1^), then for fixed $\beta _1 + \beta _2$, $R_0$ is independent of the individual values of $\beta _1$ and $\beta _2$. However, if $p_1T_{01} > p_2T_{02}$ (the case where $p_1 = 0.63, p_2 = 0.07$ TCID_50_cell^-1^d^-1^), then $R_0$ will increase with increasing $\beta _1$ (and decreasing $\beta _2$ to ensure $\beta _1 + \beta _2$ is fixed), and vice versa. Results when considering the AUC are qualitatively similar, although there are clear (but only quantitative) differences due to the non-linear dynamics of the full viral dynamics system which affect the AUC but not the calculation of $R_0$.

Then, we use the model to predict how fitness-related summary statistics change with cell tropism when the cell type with a higher viral production rate also has a higher interferon production rate (Fig. 4B). We find that there are three possible behaviours for the change in AUC with respect to cell tropism (bottom panel). The first is that the increase in virus production for cell type 1 outweighs the increase in interferon production, so preferentially infecting cell type 1 increases the AUC. For example, this happens for $p_1 = 0.385, p_2 = 0.315$ TCID_50_cell^-1^d^-1^, $q_1 = 1.25 \times 10^{-4}, q_2 = 7.5 \times 10^{-5} u_F\textrm{cell}^{-1}\textrm{d}^{-1}$ (red line in Fig. 4B). The second is that the increase in interferon production for cell type 1 outweighs the increase in virus production, so preferentially infecting cell type 2 increases the AUC. This happens for $p_1 = 0.385, p_2 = 0.315$ TCID_50_cell^-1^d^-1^, $q_1 = 1.5 \times 10^{-4}, q_2 = 5 \times 10^{-5} u_F\textrm{cell}^{-1}\textrm{d}^{-1}$ (green line in Fig. 4B). The last is that the AUC changes non-monotonically with respect to cell type preference, and is highest for an intermediate value of cell tropism. This happens for $p_1 = 0.385, p_2 = 0.315$ TCID_50_cell^-1^d^-1^, $q_1 = 1.75 \times 10^{-4}, q_2 = 2.5 \times 10^{-5} u_F\textrm{cell}^{-1}\textrm{d}^{-1}$ (blue line in Fig. 4B). However, in all three of these cases, the within-host $R_0$ increases with an increased preference for cell type 1, as changing $q_i$ does not affect $R_0$.

### For Models Lacking Innate and Adaptive Immunity, Changes in Transmissibility Due to Changes in Cell Tropism are Sensitive to the Mathematical form of the Summary Statistic

**Fig. 5:**
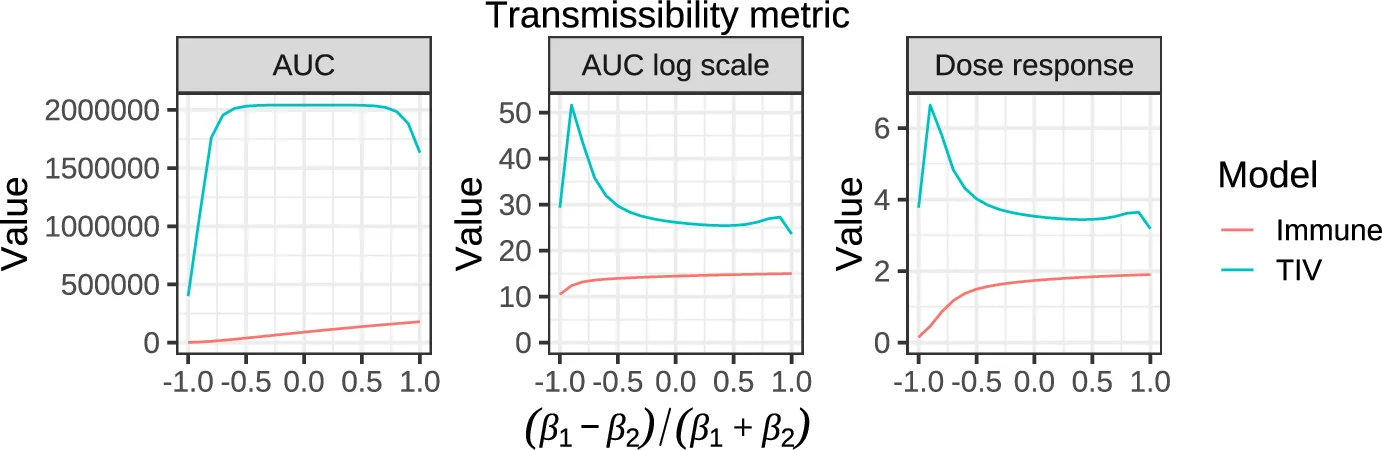
$(\beta _1 - \beta _2)/(\beta _1 + \beta _2)$ (x-axis) vs. values of transmissibility (y-axis), for (top row) a model with innate and adaptive immunity, and (bottom row) the TIV model. The summary statistics are (from left to right) $\int _{0}^{\infty } V(t) dt$, $\int _{0}^{\infty } \log _{10} V(t) dt$, and $\int _{0}^{\infty } 1 - \exp (-\gamma V(t)) dt$, using $\gamma = 10^{-4}$

Previous studies have used different functions to map between the viral load and transmissibility (see comparison in Asher et al. (2023)). We repeated the analysis in Scenario A of Sect. 3.1, but with two alternative functions for the mapping instead of the area under the viral load curve (AUC) in linear space ($\int _{0}^{\infty } V(t) dt$) which was originally used. The first is the area under the viral load curve in log space, $\int _{0}^{\infty } \log _{10} V(t) dt$. The second is the area under a dose–response transform of the viral load where transmissibility saturates with increasing viral load, $\int _{0}^{\infty } 1 - \exp (-\gamma V(t)) dt$, using $\gamma = 10^{-4}$. The parameter values were $T_{01} = 5.6 \times 10^7$, $T_{02} = 1.4 \times 10^7$, and all other parameters equal between cell types as per Table 1. Figure 5 (red lines) shows the values of each summary statistic as $\beta _1 - \beta _2$ is varied, and cell type 1 is more abundant. Monotonicity is maintained regardless of the summary statistic chosen: each summary statistic increases as $\beta _1 - \beta _2$ increases.

We repeated the analysis examining three different forms for the relationship between the viral load and transmissibility, but for a model with no innate and adaptive immunity, i.e. the TIV model found commonly in the viral dynamics literature (Baccam et al. 2006). We reduced the model in Eq. 2 to the TIV model by setting A(0)=0 and $q_i = 0$. For this model, Fig. 5 (blue lines) shows that the AUC in linear space mostly increases as $\beta _1 - \beta _2$ increases, while the AUC in log space and the area under the dose-response transformed viral load curve mostly decrease (with some non-monotonicity towards extreme values of $\beta _1 - \beta _2$).

**Fig. 6:**
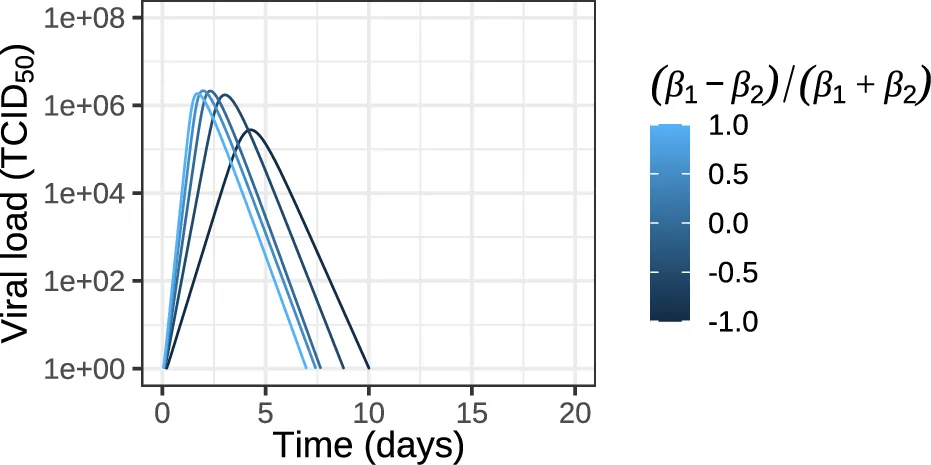
Viral load curves for the TIV model when $T_{01}/T_0 = 0.8$, as $\beta _1 - \beta _2$ is changed while $\beta _1 + \beta _2$ is held constant

To investigate further, we plot the viral load trajectories for the TIV model (Fig. 6). As $\beta _1 - \beta _2$ increases, the initial growth rate increases, leading to a faster, higher peak time, but also a much faster resolution of the infection. In the TIV model, resolution of the infection is driven by target cell depletion, so a higher initial viral growth rate leads to faster target cell depletion and a faster resolution of the infection. Comparing the three summary statistics, the AUC in log space and the area under the dose-response transformed viral load curve are more heavily influenced by the duration of the infection whereas the AUC in linear space is more heavily influenced by the peak viral load. Hence, the summary statistics’ changes in response to a viral load with a higher peak titre and shorter duration are different. For the model with innate and adaptive immune responses, the resolution of infection is due to the adaptive immune response and not target cell depletion, so the duration of infection is less sensitive to changes in cell susceptibility (Fig. 2A). Thus, increases in $\beta _1 - \beta _2$ increase all summary statistics.

### Parameters Which Were Heterogeneous Between Compartments Could be Estimated With the Right Type of Data and Knowledge of Other Parameter Values

So far, we have used models to qualitatively predict how changing cell tropism changes fitness-related summary statistics. However, to use the models to make quantitative predictions about viruses of biological interest using experimental data, we need to be able to estimate model parameters, in particular the amount of heterogeneity between cells.

To investigate whether the values of model parameters can be estimated and used to make useful predictions, we conducted a simulation estimation study. We used the model with heterogeneity in $\beta _i$ and $T_{0i}$ only, and N=2 as an example. We estimated $\beta _1$ and $\beta _2$ only. In practice, many other parameters for viral dynamics models are likely to be unidentifiable from available data (Tuncer et al. 2025), requiring alternative parameterisation strategies such as constraining parameters to values found in the literature. Here we focus on the identifiability of the parameters which are heterogeneous between cell types, and thus assume that all other parameters are known. We assume that $T_{0i}$ were known as the initial distribution of cell types is readily measurable using common techniques such as flow cytometry.

**Fig. 7:**
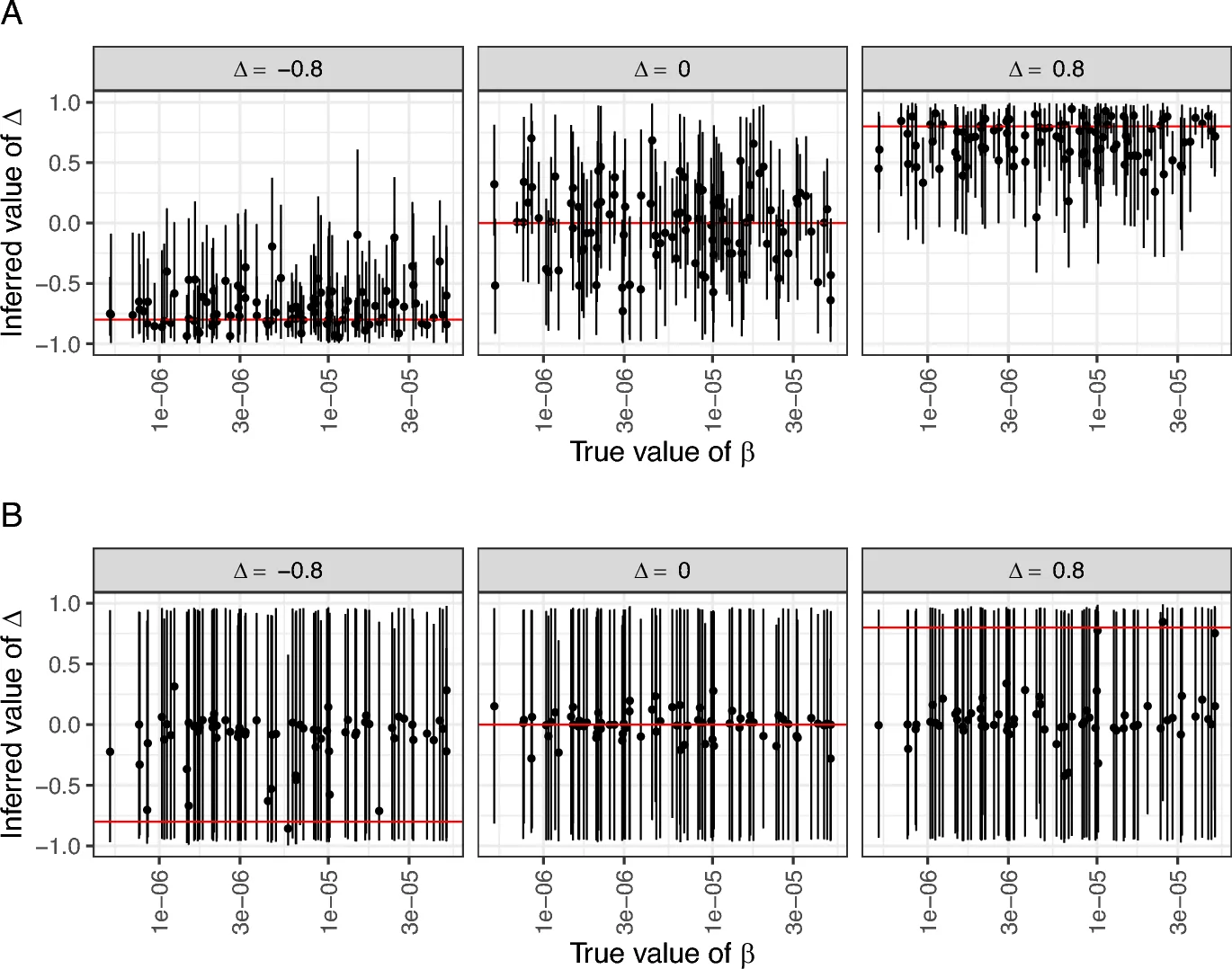
Median (dots) and 95% credible intervals (error bars) for Δ for different true values of $\bar{\beta }$ (x-axis) and Δ (top captions). Values in **A** were inferred with viral load and cell proportion data; values in **B** were inferred with viral load data only

We reparameterised $\beta _1$ and $\beta _2$ in terms of the weighted mean $\bar{\beta }$ and a parameter summarising heterogeneity in susceptibility between cell types, Δ (Methods). We simulated 100 sets of parameters varying $\bar{\beta }$, δ, p/δ, *c*, $V_0$ and $T_{01}/T_0$ (Methods). For each parameter set we simulated data using Δ=-0.8, Δ=0 and Δ=0.8 (heterogeneity is maximal when $\Delta = \pm 1$; it is difficult to establish reasonable values of Δ based on prior knowledge, as detailed in the Discussion). We then re-estimated $\bar{\beta }$ and Δ from either viral load data alone, or data on the proportion of uninfected and infected cells of each type over time in addition to viral load data. First, we assume that parameters other than $\bar{\beta }$ and Δ are known, in order to keep the focus on parameters that reflect heterogeneity. In this case, Δ could be estimated with both viral load and cell proportion data. Figure 7A shows the median (dots) and 95% credible intervals (error bars) for Δ for different true values of $\bar{\beta }$ (x-axis) and Δ (top captions), when $\bar{\beta }$ and Δ were inferred with viral load and cell proportion data. When the true value of Δ was -0.8, heterogeneity could be detected 83% of the time (that is, 83% of simulation–estimation studies recovered 95% credible intervals (CIs) for Δ which excluded 0). When the true value of Δ was 0.8, heterogeneity could be detected 75% of the time. We also tested whether there were false positives, i.e., heterogeneity was inferred even when the true value of Δ was zero. Only 3.5% of simulation–estimation studies where the true value of Δ was zero had 95% CIs for Δ excluding zero, so the rate of false positives was low. When we repeated the inference with viral load data only (Fig. 7B), the credible intervals spanned the prior distribution for Δ (uniform between -1 and 1), showing that Δ was not identifiable.

The result that data on cell proportion improve inference of Δ makes sense because if we have measurements of the relative numbers of infected cells in each compartment, these numbers reflect the relative susceptibility of these cell types of infection, whereas from the total viral load, the proportions of virus produced by each cell type are unknown.

Where individual model parameters are not identifiable (by examining the marginal posterior distribution), the joint posterior distribution may nonetheless be constrained enough to make precise model predictions. These predictions may be more important than the identifiability of model parameters themselves, for example if the main purpose of the study is to predict the impact of pharmaceutical interventions. We thus tested whether the difference in the identifiability of Δ with or without cell proportion data impacts the model’s ability to predict the viral load for hypothetical anatomical sites of infection with different initial proportions of cell types. To make these model predictions, we sampled from the joint posterior distribution of $\bar{\beta }$ and Δ and used Eq. 8 to calculate $\beta _i$. Then, holding these values of $\beta _i$ constant, we varied $T_{0i}$ while keeping their sum constant, and solved Eq. 2 to predict the viral load.

**Fig. 8:**
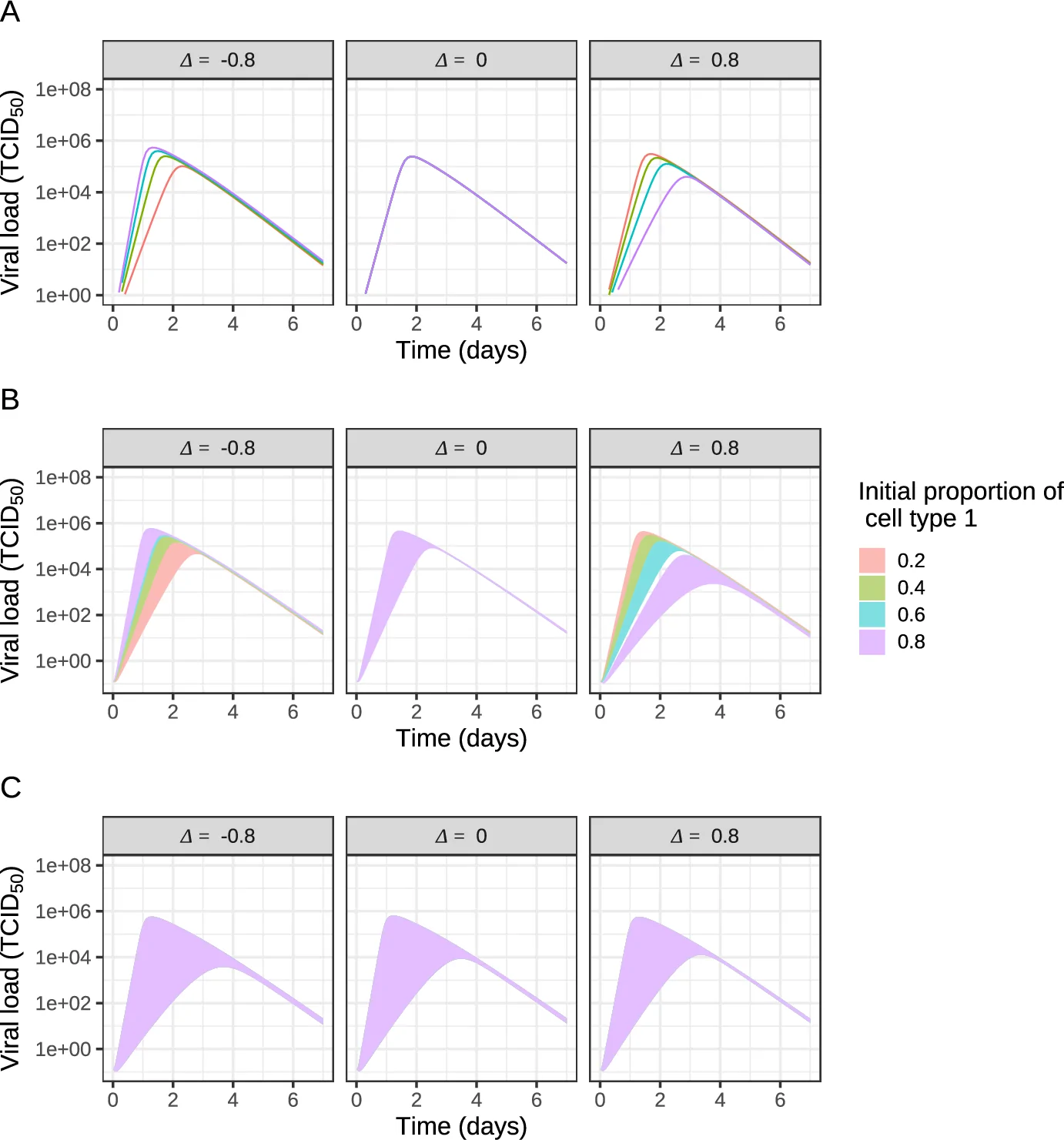
**A** Model predictions for the viral load using parameter set 1, varying the initial proportion of cell type 1 (colours) and Δ (columns) while keeping $\beta _i$ and all other parameters constant at their true values. **B**–**C** 95% credible intervals for model predictions using samples from the posterior distribution for $\beta _i$ rather than using the true values for $\beta _i$. Predictions in **B** were made using the posterior distribution inferred with viral load and cell proportion data; predictions in **C** were made using the posterior distribution inferred with viral load data only

Figure 8 shows the predictions, according to the true and fitted model parameters, for how the viral load changes as the initial cell type proportion is changed. In Fig. 8A, the lines show the model predictions for the viral load using parameter set 1, varying the initial proportion of cell type 1 (colours) and Δ (columns). When Δ=0, the true susceptibilities of the two cell types are equal, so the true model parameters predict no change in viral load regardless of the initial cell type proportion. When Δ=-0.8, then the susceptibility of cell type 1 to infection is higher than that of cell type 2 ($\beta _1> \beta _2$). Therefore, increasing the initial proportion of cell type 1 increases the initial viral growth rate and peak viral load according to the true model parameters (left column of Fig. 8A). The opposite is true when Δ=0.8. In this case, the susceptibility of cell type 2 to infection is higher than that of cell type 1 ($\beta _2 > \beta _1$). Therefore, increasing the initial proportion of cell type 1 decreases the initial viral growth rate and peak viral load according to the true model parameters (right column of Fig. 8A).

Figure 8B shows model predictions for the same initial cell type proportions as Fig. 8A, but made using parameter sets sampled from the posterior distribution, when viral load and cell proportion data (generated using the true values of Δ in the column labels) were used for model fitting. The shaded areas show the 95% credible intervals. The model fitted to the data generated assuming no cell type preference (Δ=0) correctly predicts that the viral load is independent of the initial cell type proportions. It also correctly predicts from the data generated with a preference for cell type 1 (Δ=-0.8) that the growth rate and peak viral load would increase with higher proportions of cell type 1 in this situation, and vice versa for the data generated with a preference for cell type 2 (Δ=0.8). Figure 8C shows predictions made using the posterior distribution, when only viral load data was used for fitting. The 95% credible intervals are wide and overlap for all values of $T_{01}$, so the fitted model was unable to predict the effect of changing the initial proportion of cell types.

To see whether these results are representative of the many simulated data sets used to re-estimate model parameters, Figures 2–11 in the Supplementary Material show these results for 10 of the 100 simulated data sets, and for individual parameter sets sampled from the posterior distribution rather than the 95% credible interval. Generally, in the cases where the 95% CI was able to infer heterogeneity in $\beta _i$, model predictions from individual samples reflected trends shown in the true values, while in the cases where the 95% CI was not able to infer heterogeneity in $\beta _i$, model predictions using the posterior distribution were less accurate.

We found that the degree of heterogeneity in cell types’ susceptibility to infection (Δ) could be estimated accurately with viral load and cell proportion data, assuming that parameters other than Δ and $\bar{\beta }$ (weighted average susceptibility) were known. However, many parameters in viral dynamics models are not identifiable (Tuncer et al. 2025). To test whether the practical identifiability of other parameters affects identifiability of Δ, we repeated the analysis with *p* (the virus production rate) and δ (the infected cell death rate) as additional fitted parameters. Figure 12A in the Supplementary Material shows that Δ is still able to be estimated with these additional fitted parameters, and Figure 12B in the Supplementary Material shows that model predictions with the fitted values are qualitatively similar to those made with the true parameter values. This result intuitively makes sense because Δ is informed by the proportions of infected cells of each type, and these proportions are likely to be less sensitive to *p* and δ compared to β. Thus, the result that the degree of heterogeneity in β can be estimated from viral load and cell proportion data is robust to different degrees of certainty about values of *p* and δ.

## Discussion

In this study, we constructed mathematical models for acute viral infection within the host which incorporated between-cell-type heterogeneity in susceptibility to infection, rates of virus production, and/or rates of interferon production. We have shown that when there was heterogeneity in rates of virus production and/or rates of interferon production, then summary statistics contributing to a virus’ fitness change depending on its cell tropism, i.e. which cell types are preferentially infected. In the case where there is a single source of heterogeneity, summary statistics which are expected to increase viral fitness are highest when the virus strongly preferentially infects the cell type that has the highest level of virus production or lowest level of interferon production. In the case where there are multiple competing sources of heterogeneity, the cell tropism for which the summary statistics are expected to increase fitness depends on the balance between these competing sources.

The within-host basic reproduction number only changes as heterogeneity is introduced in cells’ susceptibility to infection, rates of virus production and initial abundance, and does not change when rates relating to the innate immune response are changed. Moreover, where there is heterogeneity in cells’ rates of virus production and/or initial abundance, the basic reproduction number changes linearly with respect to changes in the amount of heterogeneity in cell types’ susceptibility to infection. On the other hand, when considering the virus’ ability to transmit to another host, we use total virus shedding as a fitness-related summary statistic. This summary statistic is affected by the innate immune response in addition to susceptibility and virus production. It can also change non-monotonically when there are multiple sources of heterogeneity, such that the highest virus shedding results from an intermediate difference between susceptibilities of cell types.

We tested three mathematical forms for the relationship between the viral load curve and transmissibility, and found that model predictions of the change in transmissibility when heterogeneity in cells’ susceptibility to infection changed were qualitatively similar regardless of the mathematical form used. However, when the TIV model was used instead of a model with innate and adaptive immunity, then two of the three mathematical forms predicted that a stronger preference for infecting more abundant cells, while increasing the within-host basic reproduction number, would ultimately decrease transmissibility through reducing the duration of infection. A similar result holds true for the TIV model even in the absence of heterogeneity: increasing β and/or *p* increases the within-host basic reproduction number while decreasing the duration of infection. To the best of the authors’ knowledge, there is no evidence for such a negative correlation between a virus’ within-host basic reproduction number and its duration of infection, or between a virus’ within-host basic reproduction number and population-level reproduction number (a measure of transmissibility). Conversely, animal studies have shown a positive correlation between an early peak in viral titre (higher basic reproduction number) and transmissibility as measured by the number of infected contact animals (Kieran et al. 2024). Thus, while the TIV model is useful for many contexts such as estimating a viral load curve with limited measurements, predictions made by varying its model parameters may not be accurate even at a qualitative level.

Two previous studies have constructed mathematical models of infection with two target cell populations. In the first of these studies (Dobrovolny et al. 2010), the parameter values for the primary cell type were held constant, while the parameter values for the secondary cell type were changed. However, when a virus evolves to infect different cell types, it is more likely that its characteristics (and so modelled parameter values) for both cell types will change simultaneously. In the second study (Reperant et al. 2012), the model was highly detailed, with compartments and parameterisations specifically for the influenza context. The study did not focus on the general qualitative behaviour of models featuring multiple target cell populations. While these models addressed the specific research questions posed by those studies, our analysis tackles the knowledge gaps of (a) how different sources of heterogeneity between cells affect within-host replication and transmissibility, and (b) the feasibility of estimating parameters associated with cell tropism from data.

This study has a number of limitations. The greatest limitation is that in the simulations to explore model behaviour, it is difficult to know what realistic values are for the degree of cell tropism and other sources of heterogeneity. Previous studies of cell tropism have either treated tropism as a binary property, where cells either do or do not have the receptors necessary for virus binding (Hikmet et al. 2020), or have treated cell tropism as a qualitative property, noting a higher or lower proportion of infected cells for different cell types (Scull et al. 2009). As an alternative, flow cytometry or single-cell RNAseq data could be used to better quantify the proportion of each infected cells for each cell type. However, if there are sources of heterogeneity between cells other than unequal susceptibilities, then the proportions of infected cells may not directly correspond to differences in susceptibilities. Thus, mathematical models to estimate the differences between cell types would be required. In the simulation-estimation study, we used either equal susceptibilities to infection or a nine-fold difference in susceptibility ($\Delta = \pm 0.8$). Once these models have been calibrated to data, then we could repeat the analysis with higher confidence. The result that the within-host basic reproduction number only changes as heterogeneity is introduced in cells’ susceptibility to infection, rates of virus production and initial abundance, and does not change when rates relating to the innate immune response are changed, is due to the model structure and is insensitive to the choice of parameter values. However, other results in the study could be sensitive to the choice of parameter values.

We focused on introducing heterogeneity to one baseline viral dynamics model for simplicity, but it is plausible that the relationship between between-cell-type heterogeneity and fitness-related summary statistics could be qualitatively different for different model formulations. Mechanisms which were absent from our model include target cell natural death and (re)growth; production of non-infectious virus, such as defective interfering particles; and natural killer cells and CD8+ T cells. However, the formulae for the basic reproduction number and growth rate are insensitive to different models of the adaptive immune response. Mathematical models with more components are also more likely to have unidentifiable parameters when fitted to limited data. Thus, the realism of the baseline model must be balanced with the data available. The sensitivity of results to baseline parameter values could also be explored systematically in future work.

We did not consider the case where the infected cell lifetime varied between cell types. This was to keep the generation time constant regardless of heterogeneity. If the generation time remains constant as the amount of heterogeneity is changed, then there is a monotonic relationship between $R_0$ and the growth rate *r*. Therefore, all results pertaining to $R_0$ will also be true for *r*. However, if the infected cell lifetime varies between cell types, then introducing heterogeneity can vary the generation time, and results which are true for $R_0$ may no longer be true for *r*. If this is the case, then more care has to be taken when comparing within-host replication summary statistics. For example, if one virus has a higher within-host $R_0$ compared to another virus but a lower value of *r*, it is not immediately clear which virus will be fitter in the full life cycle, as the virus which replicates more quickly may gain an evolutionary advantage despite producing fewer secondary virions per generation.

This study captured heterogeneity between cells in terms of their “cell type” i.e. phenotypic characteristics. However, further heterogeneity could arise spatially. Spatial heterogeneity arises partly because of both short-range and long-range modes of spread for respiratory viruses (Zeng et al. 2022), rather than the system being well-mixed as assumed by the compartmental model approach. Further heterogeneity could arise if the cell types were not distributed randomly, but according to some spatial structure. For example, influenza A virus buds from the apical surface of cells (Boulan and Sabatini 1978), in proximity to goblet cells and ciliated cells but far from basal cells which are attached to the basement membrane. The effect of the spatial location of different cell types on viral fitness, in addition to intrinsic differences, could be explored in future work using a variety of methods for spatially structured models (Williams et al. 2025).

Moreover, heterogeneity could be present even within discrete cell types. Russell et al. (2018) showed that when identical cells from an immortalised cell line were infected with influenza virus, there was extreme heterogeneity in the amount of virus produced per cell. The effect of this within-cell-type heterogeneity could be explored with partial differential equation models.

Challenges remain in transforming data into formats which are suitable to fitting of mathematical models. For example, there is no single marker for resistant cells, so identification of these cells would require combining information from the expression of multiple genes or proteins.

## Data Availability

Only simulated data are used in the study. Code to reproduce all results in the study, and all simulated data, can be found at https://github.com/ada-w-yan/fluheterogeneitypaper.

## References

[CR1] Asher J, Lemenuel-Diot A, Clay M, Durham DP, Mier-y-Teran-Romero L, Arguello CJ, Jolivet S, Wong DY, Kuhlbusch K, Clinch B, Charoin J-E (2023) Novel modelling approaches to predict the role of antivirals in reducing influenza transmission. PLoS Comput Biol 19(1):1010797. 10.1371/journal.pcbi.1010797. Publisher: Public Library of Science PMC9876374 36608108

[CR2] Baccam P, Beauchemin C, Macken CA, Hayden FG, Perelson AS (2006) Kinetics of Influenza A Virus Infection in Humans. J Virol 80(15):7590–7599. 10.1128/jvi.01623-05. Publisher: American Society for Microbiology PMC1563736 16840338

[CR3] Bajaria SH, Webb G, Cloyd M, Kirschner D (2002) Dynamics of Naive and Memory CD4+ T Lymphocytes in HIV-1 Disease Progression. JAIDS J Acquir Immune Defic Syndr 30(1):41 10.1097/00042560-200205010-00006 12048362

[CR4] Boulan ER, Sabatini DD (1978) Asymmetric budding of viruses in epithelial monlayers: a model system for study of epithelial polarity. Proc Natl Acad Sci 75(10):5071–5075. 10.1073/pnas.75.10.5071. Publisher: Proceedings of the National Academy of Sciences PMC336265 283416

[CR5] Cao P, Yan AWC, Heffernan JM, Petrie S, Moss RG, Carolan LA, Guarnaccia TA, Kelso A, Barr IG, McVernon J, Laurie KL, McCaw JM (2015) Innate Immunity and the Inter-exposure Interval Determine the Dynamics of Secondary Influenza Virus Infection and Explain Observed Viral Hierarchies. PLoS Comput Biol 11(8):1004334. 10.1371/journal.pcbi.1004334. Publisher: Public Library of Science PMC4540579 26284917

[CR6] Carnell R (2024) LHS: Latin Hypercube Samples. https://CRAN.R-project.org/package=lhs

[CR7] Dahari H, Feliu A, Garcia-Retortillo M, Forns X, Neumann AU (2005) Second hepatitis C replication compartment indicated by viral dynamics during liver transplantation. J Hepatol 42(4):491–498. 10.1016/j.jhep.2004.12.017 15763335

[CR8] Diekmann O, Heesterbeek JAP, Roberts MG (2010) The construction of next-generation matrices for compartmental epidemic models. J R Soc Interface 7(47):873–885. 10.1098/rsif.2009.0386 PMC2871801 19892718

[CR9] Dobrovolny HM, Baron MJ, Gieschke R, Davies BE, Jumbe NL, Beauchemin CAA (2010) Exploring cell tropism as a possible contributor to Influenza infection severity. PLoS ONE 5(11):13811. 10.1371/journal.pone.0013811. Publisher: Public Library of Science PMC2990709 21124892

[CR10] Elaiw AM (2010) Global properties of a class of HIV models. Nonlinear Anal Real World Appl 11(4):2253–2263. 10.1016/j.nonrwa.2009.07.001

[CR11] Fiege JK, Thiede JM, Nanda HA, Matchett WE, Moore PJ, Montanari NR, Thielen BK, Daniel J, Stanley E, Hunter RC, Menachery VD, Shen SS, Bold TD, Langlois RA (2021) Single cell resolution of SARS-CoV-2 tropism, antiviral responses, and susceptibility to therapies in primary human airway epithelium. PLoS Pathog 17(1):1009292. 10.1371/journal.ppat.1009292. Publisher: Public Library of Science PMC7872261 33507952

[CR12] FitzJohn R (2024) Odin: ODE generation and integration. https://github.com/mrc-ide/odin

[CR13] Gabry J, Češnovar R, Johnson A, Bronder S (2025) Cmdstanr: R Interface to ’CmdStan’. https://mc-stan.org/cmdstanr/

[CR14] Handel A Jr, I.M.L., Antia, R. (2007) Neuraminidase inhibitor resistance in Influenza: assessing the danger of its generation and spread. PLoS Comput Biol 3(12):240. 10.1371/journal.pcbi.0030240. Publisher: Public Library of Science PMC2134965 18069885

[CR15] Higgins D, Looker J, Sunnucks R, Carruthers J, Finnie T, Keeling MJ, Hill EM (2025) Introducing a framework for within-host dynamics and mutations modelling of H5N1 influenza infection in humans. J R Soc Interface 22(228):20240910. 10.1098/rsif.2024.0910. Publisher: Royal Society PMC12212982 40592463

[CR16] Hikmet F, Méar L, Edvinsson A, Micke P, Uhlén M, Lindskog C (2020) The protein expression profile of ACE2 in human tissues. Mol Syst Biol 16(7):209610 10.15252/msb.20209610 PMC7383091 32715618

[CR17] Kelly JN, Laloli L, V’kovski P, Holwerda M, Portmann J, Thiel V, Dijkman R (2022) Comprehensive single cell analysis of pandemic influenza A virus infection in the human airways uncovers cell-type specific host transcriptional signatures relevant for disease progression and pathogenesis. Front Immunol **13** 10.3389/fimmu.2022.978824 PMC9576848 36268025

[CR18] Kieran TJ, Sun X, Maines TR, Belser JA (2024) Optimal thresholds and key parameters for predicting influenza A virus transmission events in ferrets. npj Viruses 2(1), 64. 10.1038/s44298-024-00074-w. Publisher: Nature Publishing Group PMC11628394 39664046

[CR19] Lindeboom RGH, Worlock KB, Dratva LM, Yoshida M, Scobie D, Wagstaffe HR, Richardson L, Wilbrey-Clark A, Barnes JL, Kretschmer L, Polanski K, Allen-Hyttinen J, Mehta P, Sumanaweera D, Boccacino JM, Sungnak W, Elmentaite R, Huang N, Mamanova L, Kapuge R, Bolt L, Prigmore E, Killingley B, Kalinova M, Mayer M, Boyers A, Mann A, Swadling L, Woodall MNJ, Ellis S, Smith CM, Teixeira VH, Janes SM, Chambers RC, Haniffa M, Catchpole A, Heyderman R, Noursadeghi M, Chain B, Mayer A, Meyer KB, Chiu C, Nikolić MZ, Teichmann SA (2024) Human SARS-CoV-2 challenge uncovers local and systemic response dynamics. Nature 631(8019):189–198. 10.1038/s41586-024-07575-x. Publisher: Nature Publishing Group PMC11222146 38898278

[CR20] Lipsitch M, Barclay W, Raman R, Russell CJ, Belser JA, Cobey S, Kasson PM, Lloyd-Smith JO, Maurer-Stroh S, Riley S, Beauchemin CA, Bedford T, Friedrich TC, Handel A, Herfst S, Murcia PR, Roche B, Wilke CO, Russell CA (2016) Viral factors in influenza pandemic risk assessment. Elife 5:18491. 10.7554/eLife.18491 Publisher: eLife Sciences Publications, Ltd. PMC5156527 27834632

[CR21] Long JS, Giotis ES, Moncorgé O, Frise R, Mistry B, James J, Morisson M, Iqbal M, Vignal A, Skinner MA, Barclay WS (2016) Species difference in ANP32A underlies influenza A virus polymerase host restriction. Nature 529(7584):101–104. 10.1038/nature16474. Publisher: Nature Publishing Group PMC4710677 26738596

[CR22] Miao H, Hollenbaugh JA, Zand MS, Holden-Wiltse J, Mosmann TR, Perelson AS, Wu H, Topham DJ (2010) Quantifying the early immune response and adaptive immune response kinetics in mice infected with influenza A virus. J Virol 84(13):6687–6698. 10.1128/jvi.00266-10. Publisher: American Society for Microbiology PMC2903284 20410284

[CR23] Pawelek KA, Huynh GT, Quinlivan M, Cullinane A, Rong L, Perelson AS (2012) Modeling within-host dynamics of influenza virus infection including immune responses. PLoS Comput Biol 8(6):1002588. 10.1371/journal.pcbi.1002588. Publisher: Public Library of Science PMC3386161 22761567

[CR24] Payne RJ, Nowak MA, Blumberg BS (1992) Analysis of a cellular model to account for the natural history of infection by the hepatitis B virus and its role in the development of primary hepatocellular carcinoma. J Theor Biol 159(2):215–240. 10.1016/s0022-5193(05)80703-9 1338219

[CR25] Perelson AS, Essunger P, Cao Y, Vesanen M, Hurley A, Saksela K, Markowitz M, Ho DD (1997) Decay characteristics of HIV-1-infected compartments during combination therapy. Nature 387(6629):188–191. 10.1038/387188a0. Publisher: Nature Publishing Group 9144290

[CR26] R Core Team: R (2024) A Language and Environment for Statistical Computing. R Foundation for Statistical Computing, Vienna, Austria. https://www.R-project.org/

[CR27] Reperant LA, Kuiken T, Grenfell BT, Osterhaus ADME, Dobson AP (2012) Linking influenza virus tissue tropism to population-level reproductive fitness. PLoS ONE 7(8):43115. 10.1371/journal.pone.0043115. Publisher: Public Library of Science PMC3429484 22952637

[CR28] Roach SN, Shepherd FK, Mickelson CK, Fiege JK, Thielen BK, Pross LM, Sanders AE, Mitchell JS, Robertson M, Fife BT, Langlois RA (2024) Tropism for ciliated cells is the dominant driver of influenza viral burst size in the human airway. Proc Natl Acad Sci 121(31):2320303121. 10.1073/pnas.2320303121. Publisher: Proceedings of the National Academy of Sciences PMC11295045 39008691

[CR29] Russell AB, Trapnell C, Bloom JD (2018) Extreme heterogeneity of influenza virus infection in single cells. elife 7:32303. 10.7554/eLife.32303. Publisher: eLife Sciences Publications, Ltd. PMC5826275 29451492

[CR30] Scull MA, Gillim-Ross L, Santos C, Roberts KL, Bordonali E, Subbarao K, Barclay WS, Pickles RJ (2009) Avian Influenza virus glycoproteins restrict virus replication and spread through human airway epithelium at temperatures of the proximal airways. PLoS Pathog 5(5):1000424. 10.1371/journal.ppat.1000424. Publisher: Public Library of Science PMC2673688 19436701

[CR31] Sikkema L, Ramírez-Suástegui C, Strobl DC, Gillett TE, Zappia L, Madissoon E, Markov NS, Zaragosi L-E, Ji Y, Ansari M, Arguel M-J, Apperloo L, Banchero M, Bécavin C, Berg M, Chichelnitskiy E, Chung M-i, Collin A, Gay ACA, Gote-Schniering J, Hooshiar Kashani B, Inecik K, Jain M, Kapellos TS, Kole TM, Leroy S, Mayr CH, Oliver AJ, Papen M, Peter L, Taylor CJ, Walzthoeni T, Xu C, Bui LT, De Donno C, Dony L, Faiz A, Guo M, Gutierrez AJ, Heumos L, Huang N, Ibarra IL, Jackson ND, Kadur Lakshminarasimha Murthy P, Lotfollahi M, Tabib T, Talavera-López C, Travaglini KJ, Wilbrey-Clark A, Worlock KB, Yoshida M, Berge M, Bossé Y, Desai TJ, Eickelberg O, Kaminski N, Krasnow MA, Lafyatis R, Nikolic MZ, Powell JE, Rajagopal J, Rojas M, Rozenblatt-Rosen O, Seibold MA, Sheppard D, Shepherd DP, Sin DD, Timens W, Tsankov AM, Whitsett J, Xu Y, Banovich NE, Barbry P, Duong TE, Falk CS, Meyer KB, Kropski JA, Pe’er D, Schiller HB, Tata PR, Schultze JL, Teichmann SA, Misharin AV, Nawijn MC, Luecken MD, Theis FJ (2023) An integrated cell atlas of the lung in health and disease. Nat Med 29(6), 1563–1577. 10.1038/s41591-023-02327-2. Publisher: Nature Publishing Group PMC10287567 37291214

[CR32] Smith AP, Moquin DJ, Bernhauerova V, Smith AM (2018) Influenza virus infection model with density dependence supports biphasic viral decay. Front Microbiol **9**, 10.3389/fmicb.2018.01554. Publisher: Frontiers PMC6048257 30042759

[CR33] Stan Development Team (2025) Stan Reference Manual Version 2.37.0. https://mc-stan.org

[CR34] Tuncer N, Martcheva M, Ciupe SM (2025) Structural and practical identifiability of within-host models of virus dynamics-A review. Curr Opin Syst Biol 42:100552. 10.1016/j.coisb.2025.100552 PMC12648209 41312440

[CR35] Wang X, Song X, Tang S, Rong L (2016) Analysis of HIV models with multiple target cell populations and general nonlinear rates of viral infection and cell death. Math Comput Simul 124:87–103. 10.1016/j.matcom.2015.11.011

[CR36] Williams T, McCaw JM, Osborne JM (2025) Spatially structured models of viral dynamics: a scoping review. Microbiol Mol Biol Rev 89(4):00283–24. 10.1128/mmbr.00283-24. Publisher: American Society for Microbiology PMC12713400 40956089

[CR37] Woodall MNJ, Cujba A-M, Worlock KB, Case K-M, Masonou T, Yoshida M, Polanski K, Huang N, Lindeboom RGH, Mamanova L, Bolt L, Richardson L, Cakir B, Ellis S, Palor M, Burgoyne T, Pinto A, Moulding D, McHugh TD, Saleh A, Kilich E, Mehta P, O’Callaghan C, Zhou J, Barclay W, De Coppi P, Butler CR, Cortina-Borja M, Vinette H, Roy S, Breuer J, Chambers RC, Heywood WE, Mills K, Hynds RE, Teichmann SA, Meyer KB, Nikolić MZ, Smith CM (2024) Age-specific nasal epithelial responses to SARS-CoV-2 infection. Nat Microbiol 9(5):1293–1311. 10.1038/s41564-024-01658-1. Publisher: Nature Publishing Group PMC11087271 38622380

[CR38] Yan AWC, Zaloumis SG, Simpson JA, McCaw JM (2019) Sequential infection experiments for quantifying innate and adaptive immunity during influenza infection. PLoS Comput Biol 15(1):1006568. 10.1371/journal.pcbi.1006568. Publisher: Public Library of Science PMC6353225 30653522

[CR39] Yoshida M, Worlock KB, Huang N, Lindeboom RGH, Butler CR, Kumasaka N, Dominguez Conde C, Mamanova L, Bolt L, Richardson L, Polanski K, Madissoon E, Barnes JL, Allen-Hyttinen J, Kilich E, Jones BC, Wilton A, Wilbrey-Clark A, Sungnak W, Pett JP, Weller J, Prigmore E, Yung H, Mehta P, Saleh A, Saigal A, Chu V, Cohen JM, Cane C, Iordanidou A, Shibuya S, Reuschl A-K, Herczeg IT, Argento AC, Wunderink RG, Smith SB, Poor TA, Gao CA, Dematte JE, Reynolds G, Haniffa M, Bowyer GS, Coates M, Clatworthy MR, Calero-Nieto FJ, Göttgens B, O’Callaghan C, Sebire NJ, Jolly C, De Coppi P, Smith CM, Misharin AV, Janes SM, Teichmann SA, Nikolić MZ, Meyer KB (2022) Local and systemic responses to SARS-CoV-2 infection in children and adults. Nature 602(7896), 321–327. 10.1038/s41586-021-04345-x. Publisher: Nature Publishing Group PMC8828466 34937051

[CR40] Zeng C, Evans JP, King T, Zheng Y-M, Oltz EM, Whelan SPJ, Saif LJ, Peeples ME, Liu S-L (2022) SARS-CoV-2 spreads through cell-to-cell transmission. Proc Natl Acad Sci 119(1):2111400119. 10.1073/pnas.2111400119. Publisher: Proceedings of the National Academy of Sciences PMC8740724 34937699

